# The association between sleep duration trajectories and intrinsic capacity in middle-aged and older adults in China: a longitudinal Chinese study assessing healthy aging

**DOI:** 10.3389/fmed.2025.1595241

**Published:** 2025-07-07

**Authors:** Chengzhen Yang, Xi Chen, Xia Wan, Yinghua Cai

**Affiliations:** ^1^School of Nursing, Nanjing Medical University, Nanjing, China; ^2^The Affiliated Wuxi People’s Hospital of Nanjing Medical University, Wuxi, China; ^3^Wuxi Medical Center, Nanjing Medical University, Wuxi, China; ^4^Wuxi People’s Hospital, Wuxi, China

**Keywords:** sleep duration, sleep health, intrinsic capacity, healthy aging, GBMTM

## Abstract

**Background:**

Previous studies have focused mostly on the association between a single measurement of nighttime sleep duration and intrinsic capacity, making revealing the dynamic interaction between nighttime sleep duration and nap duration as individuals age throughout their lifespan difficult. This study aimed to identify the joint developmental trajectories of nighttime sleep duration and nap duration and explore their associations with intrinsic capacity.

**Method:**

Data from 5,618 participants in the China Health and Retirement Longitudinal Study across three waves (2011, 2013, 2015) were analyzed. Group-based multi-trajectory modeling was employed to identify joint developmental trajectories of nighttime sleep and nap duration, and a binary logistic regression analysis was used to explore the associations between joint developmental trajectories and intrinsic capacity.

**Results:**

Four distinct joint developmental trajectories were identified. Compared with the “Continuous moderate nighttime sleep without napping” trajectory group, the “Persistent short sleep at night without napping” trajectory group (OR = 1.64, 95% CI: 1.26–2.12) exhibited a significantly higher risk of low intrinsic capacity. Conversely, the “Double moderate sleep duration” trajectory group was more likely to have high intrinsic capacity (OR = 0.81, 95% CI: 0.68–0.96). No significant association was observed in the “Persistent short nighttime sleep with moderate napping” trajectory group (OR = 1.00, 95% CI: 0.82–1.21).

**Conclusion:**

Persistent short nighttime sleep patterns are significantly associated with a risk of low intrinsic capacity, whereas moderate napping may offset the adverse effects of persistent short nighttime sleep on intrinsic capacity. Conversely, a combined pattern of moderate nighttime sleep and moderate napping appears most beneficial for maintaining high intrinsic capacity. This study suggests that scientifically planning sleep duration is important for maintaining high intrinsic capacity, providing new theoretical references for optimizing the “dual-mode sleep” management strategy for middle-aged and older adults.

## 1 Background

As life expectancy increases and birth rates continue to decline, the global aging process is accelerating at an unprecedented pace. According to projections, between 2019 and 2050, the global population aged 60 and above will surge from 1 billion to 2.1 billion, with their share of the total population rising from 13 to 22% (1). The intensification of population aging poses a serious challenge to societies and healthcare systems around the world. For elderly individuals, maintaining good physical and mental health and ensuring their ability to participate freely in social activities has become a core issue in improving their quality of life (2). However, even in the absence of disease, older adults may still experience degenerative changes in physical function, psychological status, cognitive function, and other aspects related to age, which can negatively impact their life and social development (3). In view of this, to respond actively to the challenges of aging, the World Health Organization (WHO) has proposed a function-based assessment method that uses intrinsic capacity (IC) as a key indicator to measure healthy aging (4). Intrinsic capacity refers to the sum of an individual’s physical and mental abilities that can be mobilized at a given point in time, covering five core domains: locomotion, sensory capacity, vitality, cognitive capacity, and psychological capacity (5). This assessment system breaks away from the traditional disease-oriented framework of health understanding and instead focuses on the biological mechanisms of the aging process, reflecting the health status of older adults from a more fundamental level (6). Since the WHO proposed this assessment method, many empirical studies have fully verified the scientific nature and effectiveness of the intrinsic capacity structure (3, 7, 8), and its important position as a core strategy for healthy aging has been widely recognized in academic circles. Intrinsic capacity assessment provides an integrated analytical framework for systematically identifying the health needs of older adults and formulating precise intervention strategies, demonstrating enormous application potential in the field of geriatric health management (9, 10). However, research shows that the evolution of intrinsic capacity does not begin in old age but rather develops throughout the life cycle (7). Therefore, early identification of risk factors associated with intrinsic capacity decline and timely implementation of preventive interventions before significant functional impairment occurs in older adults are crucial for maintaining and improving their health and delaying the process of functional decline.

Sleep, as an important cornerstone of individual health and well-being, occupies a central position in the health management of elderly individuals (11). However, epidemiological research data show that the prevalence of sleep disorders among the elderly population worldwide remains high and that sleep quality decreases significantly with age (12–14). Long-term poor sleep patterns not only hinder successful aging in older adults (15) but are also closely associated with multiple health risk factors, including cognitive decline (16), cardiovascular disease (17), mental health problems (18), and obesity (19). Therefore, sleep health has become an important indicator for assessing the overall health and functional status of older adults (20). Previous studies have shown that poor sleep quality accelerates the decline in intrinsic capacity in older adults (21, 22), whereas maintaining a healthy pattern of 6–8 h of nighttime sleep helps delay intrinsic capacity decline (23). However, most of these studies are based on nighttime sleep duration measured at a single point in time and fail to capture the longitudinal dynamics of sleep duration with age (24). In addition, some studies have focused only on the association between nighttime sleep duration and intrinsic capacity, ignoring the potential impact of nap duration on the health of older adults (25, 26). Even when nighttime sleep and nap duration are considered together, they are often analyzed independently, failing to reveal the synergistic development between the two factors (23). Owing to the dynamic nature of sleep characteristics, a single measurement cannot fully reflect the true association between sleep duration and intrinsic capacity. Therefore, further research is needed on the dynamic development patterns of sleep duration over the life cycle and its association with intrinsic capacity.

Against the backdrop of accelerating global population aging, China’s aging situation is becoming increasingly severe (27). According to forecast data, between 2010 and 2040, the proportion of China’s population aged 60 and above will surge from 12.4 to 28%, and the trend of aging cannot be underestimated (28). China’s unique cultural background and diverse lifestyles may cause the joint developmental trajectory of nighttime sleep duration and nap duration among middle-aged and older adults in China to differ from that of Western populations, thereby producing different effects on intrinsic capacity. Therefore, this study takes middle-aged and older adults in China as the research objects and systematically investigates the impact of the joint developmental trajectory of nighttime sleep duration and nap duration on intrinsic capacity. The aim of this study is to provide a reference for medical workers to identify health risks in middle-aged and older adults at an early stage and to provide a new theoretical reference for the formulation of scientific and effective intervention strategies to increase their intrinsic capacity.

## 2 Methods

### 2.1 Study population

The data for this study come from the China Health and Retirement Longitudinal Study (CHARLS) database, which was initiated and constructed by the National Development Research Institute of Peking University. The CHARLS focuses on Chinese residents aged 45 and above and comprehensively collects multidimensional data such as basic personal information, socioeconomic status, and physical health status. This study aims to respond actively to the various challenges faced by China in the process of accelerating population aging. The baseline survey of CHARLS was officially launched in 2011, covering 28 provinces across the country, with 17,708 participants enrolled. The final response rate reached 80.5%, ensuring the national representativeness of the data through extensive sample coverage. To ensure the timeliness and continuity of the data, the research team subsequently conducted four nationwide follow-up surveys in 2013, 2015, 2018, and 2020, continuously updating the data resources. The methodological details regarding sample selection and data collection procedures have been described in detail in previous studies (29). Prior to the implementation of the study, all participants signed informed consent forms. Additionally, this study was approved by the Ethics Review Committee of Peking University (IRB 00001052–11,015) and was conducted in accordance with the Strengthening the Reporting of Observational Studies in Epidemiology (STROBE) guidelines to ensure transparency and ethical compliance.

**Figure 1:**
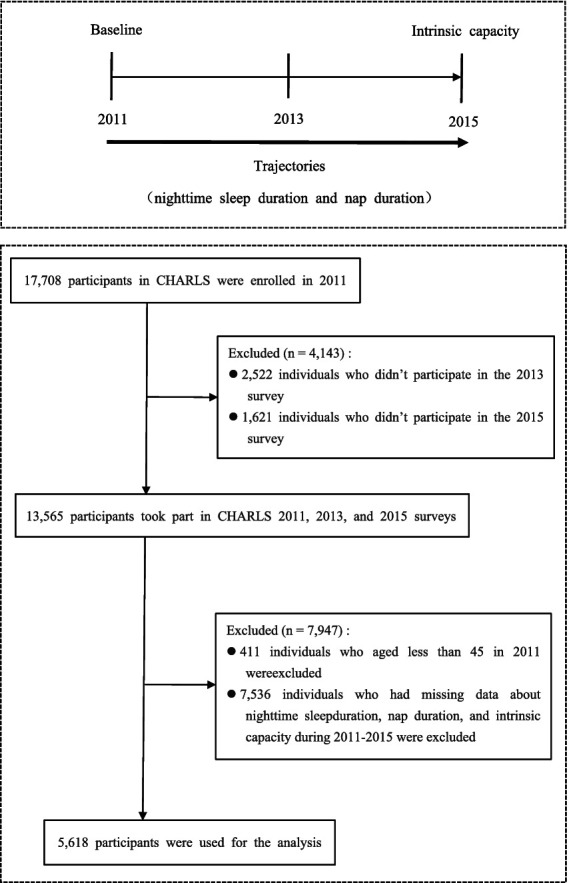
Flow diagram for participants included in the study.

Given that the CHARLS project did not assess the dimensions of intrinsic capacity related to locomotion and vitality in 2018 and 2020, this study selected data from the 2011, 2013, and 2015 surveys as the basis for the study. Before the data were analyzed, the research team cleaned the raw data to ensure their completeness and consistency. The specific steps are as follows: First, this study excluded individuals who did not participate in the 2013 and 2015 follow-up surveys. Second, this study adopted a differentiated approach to address data missingness. Participants with missing data on nighttime sleep duration, nap duration, and intrinsic capacity in the three rounds of surveys were excluded to ensure the accuracy of the basic data for analysis. For individuals with missing values in covariates, multiple imputation was used to process the data, thereby making better use of existing information and enhancing the validity of the estimates. After systematic data cleaning and screening, a total of 5,618 participants were included in the analysis. The detailed sample screening process is shown in Figure 1. The sample covers middle-aged and elderly individuals from different geographical regions, age groups, and socioeconomic backgrounds in China, ensuring the national representativeness of the analysis results and the generalizability of the research conclusions.

### 2.2 Nighttime sleep duration measurement

The participants’ nighttime sleep duration data were collected using a standardized questionnaire (15) “Over the past month, how many hours did you actually sleep each night? (average number of hours per night).” This open-ended question design avoids response bias that may arise from predefined options, ensuring that the data accurately reflect participants’ sleep patterns. The classification criteria for nighttime sleep duration were based on previous studies (23, 30), with sleep durations of less than 6 h per night defined as short sleep, durations of 6–8 h as normal sleep, and durations of more than 8 h as long sleep.

### 2.3 Nap duration measurement

In the CHARLS project, participants’ nap duration was assessed using a standardized questionnaire, which asked, “How long did you usually sleep during the day in the past month?.” On the basis of the standards recommended for the relationship between nap duration and intrinsic capacity in previous studies (23), this study divided participants into the following three groups: no nap group (nap duration of 0 min), moderate nap group (nap duration ≤ 60 min), and long nap group (nap duration > 60 min).

### 2.4 Intrinsic capacity assessment

#### 2.4.1 Locomotion

Locomotion was assessed using a five-time sit-to-stand test. The participants who were able to complete the test independently within 14 s were given a score of 1; otherwise, they were given a score of 0 (31, 32). The results of this test effectively reflect an individual’s level of limb motor function.

#### 2.4.2 Sensory capacity

Sensory capacity assessment includes vision and hearing. Vision assessment was conducted by asking, “How well can you see objects at a distance? For example, can you recognize a friend across the street (even when wearing glasses)?.” Hearing assessment was conducted by asking, “How is your hearing? (If you wear a hearing aid, how is your hearing when wearing it?).” The participants chose from five options: “excellent,” “good,” “fair,” “average,” or “poor.” Referring to previous studies (31), if participants answered “poor” to either question, they were diagnosed with a sensory capacity impairment and given a sensory capacity score of 0; if both answers were “excellent,” “good,” “fair,” or “average,” they were given a score of 1.

#### 2.4.3 Vitality

Referring to previous studies (33–35), vitality is assessed based on two dimensions: respiratory function (peak flow test) and dominant hand strength (grip strength test). The peak expiratory flow rate is calculated as the average of two measurements. The threshold value for males is 350 L/min (≤350 is 0 points, >350 is 1 point), and the threshold value for females is 220 L/min (≤220 is 0 points, >220 is 1 point). The dominant hand grip strength is also calculated as the average of two measurements. The threshold for males is 36 kg (scores below 36 are 0 points, and scores of 36 or above are 1 point), and the threshold for females is 23 kg (scores below 23 are 0 points, and scores of 23 or above are 1 point). Finally, the vitality total score is calculated by adding the scores from the two dimensions (total score 0–2 points). A score below 2 is classified as limited vitality and assigned a value of 0, while a score of 2 is classified as normal vitality and assigned a value of 1.

#### 2.4.4 Psychological capacity

Psychological capacity was assessed using 10 items from the Center for Epidemiologic Studies Depression Scale (CES-D). On the basis of the scale scores, those with CES-D scores < 10 were classified as having no depressive symptoms and assigned a value of 1; those with scores ≥ 10 were diagnosed as having depressive symptoms and assigned a value of 0, thereby completing the classification of depressive symptoms (23).

#### 2.4.5 Cognitive capacity

Cognitive capacity was assessed in four dimensions: time orientation, calculation and attention, visual–spatial, and episodic memory. The temporal orientation dimension includes five questions on year, month, day, week, and season; the calculation and attention dimension requires participants to perform five subtraction operations; the visual–spatial dimension involves drawing two overlapping pentagons; and the episodic memory dimension is assessed through immediate and delayed recall of 10 words, with each correctly recalled word scoring 1 point, and the average of the two recall scores serving as the memory score. Except for the memory section, each correct answer was given a score of 1 point, and the total cognitive capacity score was calculated by adding the scores of each question, with a score ranging from 0 to 21 points (36). Referring to previous studies (34, 37), the cognitive function scores were standardized using the Z-score standardization, with a positive Z-score indicating cognitive capacity above average and assigned a value of 1 and a negative Z-score indicating cognitive capacity below average and assigned a value of 0.

#### 2.4.6 Total intrinsic capacity score

The total intrinsic capacity score is the sum of the scores for the five dimensions of locomotion, sensory capacity, vitality, psychological capacity, and cognitive capacity, ranging from 0 to 5 points. Referring to previous studies (34), this study used Z-score standardization to convert the total intrinsic capacity score into a categorical variable. A positive Z-score corresponds to higher intrinsic capacity and is assigned a value of 0, whereas a negative Z-score corresponds to lower intrinsic capacity and is assigned a value of 1.

### 2.5 Covariates

To ensure the accuracy and reliability of the research results and effectively control for the influence of potential confounding factors, this study included covariates from three dimensions: demographic characteristics, lifestyle behaviors, and health status. The demographic characteristics collected include age, gender (female/male), marital status (others/married), residence (urban/rural), education level (primary school or below/junior high school or above), ethnic group (others/Han Chinese), and health insurance status (no/yes). The covariates related to lifestyle behaviors include smoking (no/yes), alcohol drinking (no/yes), and social participation. Health status covariates mainly included chronic diseases (including hypertension, diabetes, heart disease, cancer, stroke, mental disorder, and cognitive impairment), multimorbidity (none/1–2/3–5/≥6), self-rated health status (unhealthy/healthy), disability (no/yes), BMI (underweight/normal weight/overweight/obese), ADL, and baseline intrinsic capacity(low/high).

### 2.6 Statistical analysis

To accurately identify the joint developmental trajectory of nighttime sleep duration and nap duration, this study used the “Traj” plug-in (38) of Stata 18.0 software to construct a group-based multi-trajectory model (GBMTM) (39). As an extension of the group-based trajectory model (GBTM) (39), this model uses a limited mixture model to cluster and identify trajectory groups with different developmental patterns, thereby capturing the joint developmental trajectories of multiple variables (such as nighttime sleep duration and nap duration in this study). Model construction strictly follows the two-stage method proposed by Nagin et al. (40). In the first stage, through multiple model fittings, the optimal number of clusters is determined on the basis of indicators such as the Bayesian information criterion (BIC), average posterior probability (AvePP), and entropy value. In the second stage, polynomial functions are used to fit and characterize the curve shapes of each trajectory group. During model selection, the core criterion is to minimize the absolute value of the BIC while ensuring that the AvePP of each group is greater than 0.7, the entropy value is greater than 0.7 and approaches 1, and the sample proportion of each group is greater than 5%, thereby ensuring the scientific validity and stability of the model grouping.

Data analysis was performed using R software version 4.4.1. Quantitative data that conformed to a normal distribution were expressed as the mean ± standard deviation (M ± SD) and multiple group comparisons were performed using one-way analysis of variance (ANOVA). Quantitative data that did not follow a normal distribution were expressed as the median (M) and interquartile range (P25, P75), and group comparisons were performed using the Kruskal–Wallis H rank test. Count data were described as rates or proportions, and multiple group comparisons were performed using the chi-square test. A binary logistic regression model was used to analyze the association between sleep duration joint developmental trajectory and intrinsic capacity, and three statistical models were constructed: Model 1 was the unadjusted baseline model; Model 2 included demographic variables such as age, gender, marital status, residence, education level, ethnic group, and health insurance status in addition to Model 1; Model 3 further added confounding factors such as smoking, alcohol drinking, social participation, chronic diseases, multimorbidity, self-rated health status, disability, BMI, ADL, and baseline intrinsic capacity to gradually control for potential confounding variables, thereby enhancing the accuracy and reliability of the study results.

In order to verify the robustness of the association between joint development trajectories and intrinsic capacity, this study conducted subgroup analysis based on variables such as age, gender, residence, smoking, BMI, and multimorbidity, and explored the interaction between subgroup variables and joint development trajectories through likelihood ratio tests. In addition, to further verify the robustness of the research results, this study conducted a sensitivity analysis: (1) the intrinsic capacity indicator was converted from a categorical variable to a continuous variable, and its association with the joint development trajectory was reanalyzed; (2) inverse probability weighting (IPW) was used to adjust the potential confounding bias of the baseline characteristics and test the stability of the main analysis results, thereby improving the reliability and scientific nature of the research conclusions.

All the statistical tests in this study were performed using two-tailed tests, with a significance level of *α* = 0.05. When the test *p* value was <0.05, the results were considered statistically significant.

## 3 Results

### 3.1 Characteristics of the study population

This study included a total of 5,618 participants. Table 1 presents the baseline characteristics of the participants classified according to the joint developmental trajectories of nighttime sleep duration and nap duration. The results of the group comparison revealed that there were significant differences between the participants in each trajectory group in terms of age, gender, marital status, education level, ethnic group, smoking, alcohol drinking, hypertension, diabetes, heart disease, mental disorder, multimorbidity, self-rated health status, BMI, ADL, and intrinsic capacity (*p* < 0.05). In contrast, there were no statistically significant differences in terms of social participation, residence, health insurance, cancer, stroke, cognitive impairment, and disability (*p* > 0.05). Supplementary Table S1 shows the comparison of baseline characteristics between included and excluded participants. A total of 7,536 participants were excluded due to missing key data such as nighttime sleep duration, nap duration, and intrinsic capacity. Compared with participants included in the analysis, excluded participants were more likely to be male, married, have a junior high school education or higher, currently smoke and drink alcohol, and have higher intrinsic capacity.

**Table 1:** Characteristics of all participants by trajectory group.

Variables	Total (*n* = 5,618)	G1^a^ (*n* = 415)	G2^b^ (*n* = 1,526)	G3^c^ (*n* = 1,290)	G4^d^^s^ (*n* = 2,387)	Statistic	*P*
Age, mean ± SD	57.85 ± 8.18	59.13 ± 8.33	56.75 ± 7.92	58.70 ± 8.05	57.87 ± 8.30	*F* = 17.30	<0.001
Gender, *n* (%)						χ^2^ = 138.94	<0.001
Female	2,657 (47.29)	275 (66.27)	808 (52.95)	631 (48.91)	943 (39.51)		
Male	2,961 (52.71)	140 (33.73)	718 (47.05)	659 (51.09)	1,444 (60.49)		
Marital status, *n* (%)						χ^2^ = 11.22	0.011
Others	509 (9.06)	56 (13.49)	126 (8.26)	115 (8.91)	212 (8.88)		
Married	5,109 (90.94)	359 (86.51)	1,400 (91.74)	1,175 (91.09)	2,175 (91.12)		
Residence, *n* (%)						χ^2^ = 3.56	0.314
Urban	2026 (36.06)	135 (32.53)	544 (35.65)	484 (37.52)	863 (36.15)		
Rural	3,592 (63.94)	280 (67.47)	982 (64.35)	806 (62.48)	1,524 (63.85)		
Education level, *n* (%)						χ^2^ = 63.66	<0.001
Primary school or below	3,491 (62.14)	312 (75.18)	983 (64.42)	835 (64.73)	1,361 (57.02)		
Junior high school or above	2,127 (37.86)	103 (24.82)	543 (35.58)	455 (35.27)	1,026 (42.98)		
Ethnic group, *n* (%)						χ^2^ = 24.86	<0.001
Others	313 (5.57)	32 (7.71)	116 (7.60)	51 (3.95)	114 (4.78)		
Han Chinese	5,305 (94.43)	383 (92.29)	1,410 (92.40)	1,239 (96.05)	2,273 (95.22)		
Health insurance, *n* (%)						χ^2^ = 1.72	0.633
No	287 (5.11)	19 (4.58)	81 (5.31)	73 (5.66)	114 (4.78)		
Yes	5,331 (94.89)	396 (95.42)	1,445 (94.69)	1,217 (94.34)	2,273 (95.22)		
Smoking, *n* (%)						χ^2^ = 42.59	<0.001
No	3,727 (66.34)	314 (75.66)	1,041 (68.22)	891 (69.07)	1,481 (62.04)		
Yes	1891 (33.66)	101 (24.34)	485 (31.78)	399 (30.93)	906 (37.96)		
Alcohol drinking, *n* (%)						χ^2^ = 43.03	<0.001
No	3,557 (63.31)	308 (74.22)	1,015 (66.51)	814 (63.10)	1,420 (59.49)		
Yes	2061 (36.69)	107 (25.78)	511 (33.49)	476 (36.90)	967 (40.51)		
Social participation, mean ± SD	1.53 ± 1.91	1.31 ± 1.71	1.57 ± 1.97	1.48 ± 1.87	1.56 ± 1.93	*F* = 2.59	0.051
Hypertension, *n* (%)						χ^2^ = 22.44	<0.001
No	4,256 (75.76)	314 (75.66)	1,217 (79.75)	932 (72.25)	1793 (75.12)		
Yes	1,362 (24.24)	101 (24.34)	309 (20.25)	358 (27.75)	594 (24.88)		
Diabetes, *n* (%)						χ^2^ = 8.14	0.043
No	5,301 (94.36)	396 (95.42)	1,458 (95.54)	1,204 (93.33)	2,243 (93.97)		
Yes	317 (5.64)	19 (4.58)	68 (4.46)	86 (6.67)	144 (6.03)		
Cancer, *n* (%)						χ^2^ = 0.59	0.898
No	5,575 (99.23)	411 (99.04)	1,516 (99.34)	1,279 (99.15)	2,369 (99.25)		
Yes	43 (0.77)	4 (0.96)	10 (0.66)	11 (0.85)	18 (0.75)		
Heart disease, *n* (%)						χ^2^ = 18.54	<0.001
No	4,975 (88.55)	357 (86.02)	1,384 (90.69)	1,109 (85.97)	2,125 (89.02)		
Yes	643 (11.45)	58 (13.98)	142 (9.31)	181 (14.03)	262 (10.98)		
Stroke, *n* (%)						χ^2^ = 4.00	0.262
No	5,521 (98.27)	406 (97.83)	1,508 (98.82)	1,264 (97.98)	2,343 (98.16)		
Yes	97 (1.73)	9 (2.17)	18 (1.18)	26 (2.02)	44 (1.84)		
Mental disorder, *n* (%)						χ^2^ = 22.81	<0.001
No	5,551 (98.81)	406 (97.83)	1,517 (99.41)	1,261 (97.75)	2,367 (99.16)		
Yes	67 (1.19)	9 (2.17)	9 (0.59)	29 (2.25)	20 (0.84)		
Cognitive impairment, *n* (%)						χ^2^ = 4.72	0.193
No	5,558 (98.93)	411 (99.04)	1,507 (98.75)	1,271 (98.53)	2,369 (99.25)		
Yes	60 (1.07)	4 (0.96)	19 (1.25)	19 (1.47)	18 (0.75)		
Multimorbidity, *n* (%)						χ^2^ = 158.08	<0.001
None	1847 (32.88)	84 (20.24)	574 (37.61)	323 (25.04)	866 (36.28)		
1–2	2,797 (49.79)	224 (53.98)	746 (48.89)	644 (49.92)	1,183 (49.56)		
3–5	916 (16.30)	98 (23.61)	198 (12.98)	299 (23.18)	321 (13.45)		
≥6	58 (1.03)	9 (2.17)	8 (0.52)	24 (1.86)	17 (0.71)		
Self-rated health status, *n* (%)						χ^2^ = 124.24	<0.001
Unhealthy	1,252 (22.29)	162 (39.04)	292 (19.13)	363 (28.14)	435 (18.22)		
Healthy	4,366 (77.71)	253 (60.96)	1,234 (80.87)	927 (71.86)	1952 (81.78)		
Disability, *n* (%)						χ^2^ = 1.27	0.736
No	5,452 (97.05)	399 (96.14)	1,482 (97.12)	1,253 (97.13)	2,318 (97.11)		
Yes	166 (2.95)	16 (3.86)	44 (2.88)	37 (2.87)	69 (2.89)		
BMI, *n* (%)						χ^2^ = 50.58	<0.001
Underweight	308 (5.48)	38 (9.16)	96 (6.29)	73 (5.66)	101 (4.23)		
Normal weight	2,929 (52.14)	231 (55.66)	849 (55.64)	669 (51.86)	1,180 (49.43)		
Overweight	1703 (30.31)	105 (25.30)	401 (26.28)	405 (31.40)	792 (33.18)		
Obese	678 (12.07)	41 (9.88)	180 (11.80)	143 (11.09)	314 (13.15)		
ADL, Mean ± SD	0.19 ± 0.62	0.40 ± 0.94	0.14 ± 0.52	0.28 ± 0.73	0.14 ± 0.52	*F* = 34.27	<0.001
Intrinsic capacity, *n* (%)						χ^2^ = 159.72	<0.001
High	4,225 (75.20)	223 (53.73)	1,161 (76.08)	910 (70.54)	1931 (80.90)		
Low	1,393 (24.80)	192 (46.27)	365 (23.92)	380 (29.46)	456 (19.10)		

F: ANOVA, χ^2^: Chi-square test, SD: standard deviation.

a “Persistent short sleep at night without napping” trajectory group.

b “Continuous moderate nighttime sleep without napping” trajectory group.

c “Persistent short sleep at night with moderate naps” trajectory group.

d “Double moderate sleep duration” trajectory group.

### 3.2 Modeling the joint developmental trajectory of nighttime sleep duration and nap duration

**Figure 2:**
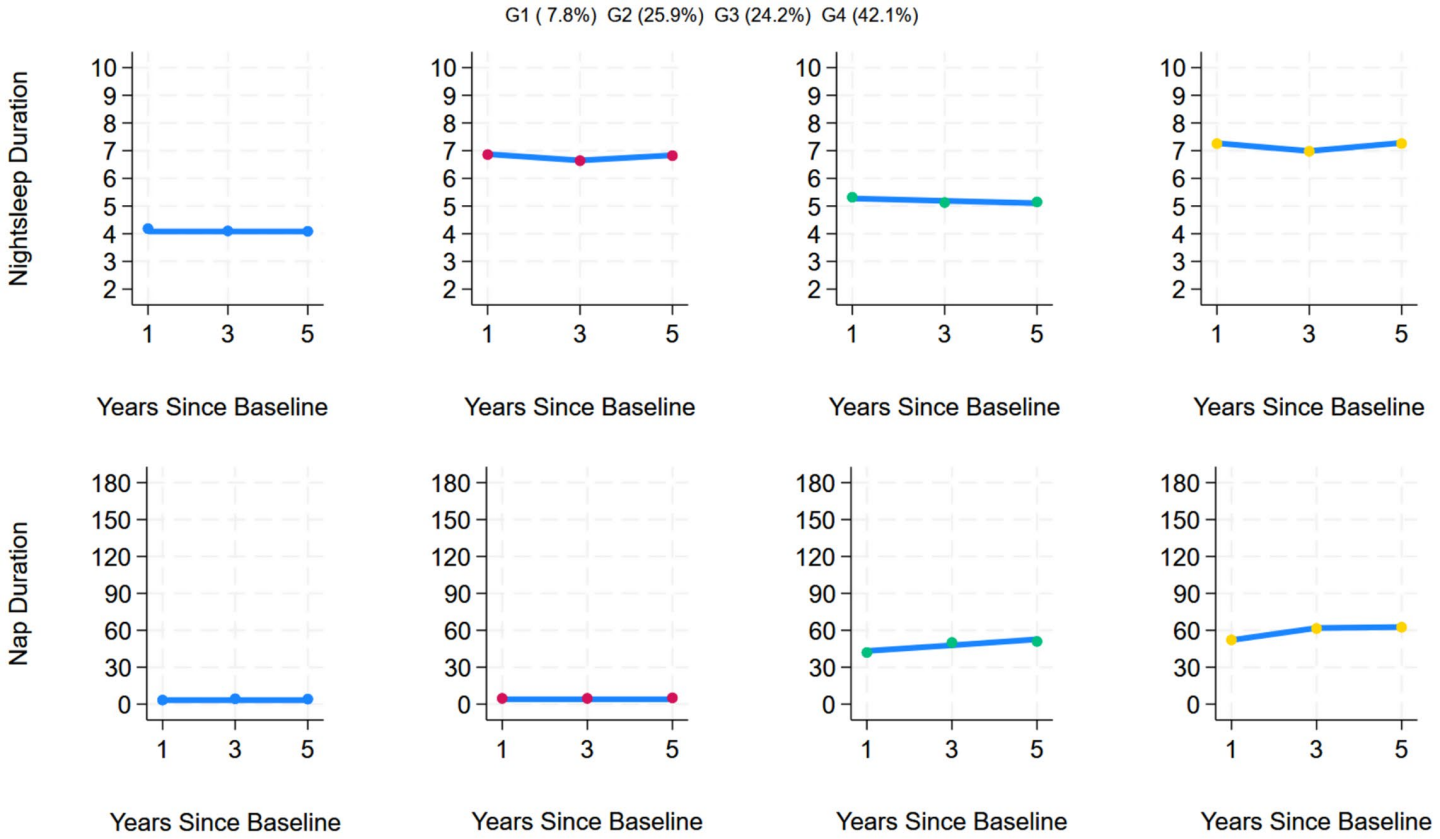
Trajectories of sleep duration among the CHARLS participants.

This study included data on nighttime sleep duration and nap duration from 2011 to 2015 and used a group-based multi-trajectory model for analysis, ultimately determining that the optimal number of trajectories was 4. During trajectory modeling, models 5 and 6 were excluded due to issues such as entropy values <0.7, AvePP <0.7, or trajectory group sample proportions <5%. Compared with the first three models, Model 4 had the smallest absolute BIC value of −91037.36, an entropy value of 0.715, AvePP > 0.7, and the proportion of samples in each group was > 5%, indicating that the trajectory division had high reliability and stability, verifying the rationality and accuracy of Model 4. The estimated values of all the parameters for the fitted trajectories are detailed in Table 2. Among the 5,618 individuals included in the study, four joint developmental trajectories of nighttime sleep duration and nap duration were identified. A total of 7.8% (*n* = 415) followed the “Persistent short sleep at night without napping” trajectory, in which older adults in this group maintained short nighttime sleep (<6 h) throughout the 5-year follow-up period and no napping habits; 25.9% (*n* = 1,526) followed the “Continuous moderate nighttime sleep without napping” trajectory, characterized by consistently moderate nighttime sleep (6–8 h) over 5 years but no napping habits; 24.2% (*n* = 1,290) followed the “Persistent short sleep at night with moderate naps” trajectory, characterized by persistent short nighttime sleep (<6 h) but maintaining a moderate napping habit (≤60 min); and 42.1% (*n* = 2,387) followed the “Double moderate sleep duration” trajectory group, characterized by maintaining moderate nighttime sleep (6–8 h) for 5 years, accompanied by moderate napping (≤60 min). Figure 2 shows the four trends of the synergistic changes in nighttime sleep duration and nap duration over time.

**Table 2:** Fit indices of the GBMTM on trajectories.

Model	aBIC	BIC	AIC	Entropy	AvePP(%)	Class (%)
1	−93879.50	−93873.23	−93850.01	1.000	1.00	100.0
2	−92178.24	−92166.59	−92123.47	0.763	0.90/0.95	34.8/65.2
3	−91363.06	−91347.83	−91291.44	0.752	0.88/0.91/0.82	25.8/55.8/18.4
4	−91037.36	−91018.54	−90948.89	0.715	0.83/0.84/0.79/0.86	7.8/25.9/24.2/42.1
5	−90884.62	−90863.12	−90783.51	0.696	0.79/0.78/0.82/0.79/0.82	12.0/18.9/4.9/34.3/29.8
6	−90813.76	−90794.05	−90721.08	0.670	0.83/0.86/0.69/0.80/0.75/0.72	8.1/18.6/15.7/6.1/37.0/14.5

GBMTM: group-based multi-trajectory model. BIC: bayesian information criterion. AvePP: average posterior probability.

### 3.3 Analysis of the associations between joint developmental trajectories and intrinsic capacity

The results of the binary logistic regression showed that, compared with the “continuous moderate nighttime sleep without napping” trajectory group, the “persistent short sleep at night without napping” trajectory group had a significantly higher risk of low intrinsic capacity (OR = 1.64, 95% CI: 1.26–2.12). The “double moderate sleep duration” trajectory group was more likely to have high intrinsic capacity (OR = 0.81, 95% CI: 0.68–0.96). However, no statistically significant association was found between the “persistent short nighttime sleep with moderate napping” trajectory group and the risk of low intrinsic capacity (OR = 1.00, 95% CI: 0.82–1.21). The specific associations between the joint developmental trajectories and intrinsic capacity are shown in Table 3.

**Table 3:** Associations between trajectory groups and intrinsic capacity.

Trajectory group	Model 1^a^ OR (95% CI)	Model 2^b^ OR (95% CI)	Model 3^c^ OR (95% CI)
Group 2	1.00 (Reference)	1.00 (Reference)	1.00 (Reference)
Group 1	2.74 (2.18, 3.43)	2.29 (1.80, 2.91)	1.64 (1.26, 2.12)
Group 3	1.33 (1.12, 1.57)	1.25 (1.04, 1.49)	1.00 (0.82, 1.21)
Group 4	0.75 (0.64, 0.88)	0.74 (0.63, 0.88)	0.81 (0.68, 0.96)

OR, Odds Ratio; CI, Confidence Interval.

a Model 1 did not adjust any variables.

b Model 2 adjusted for age, gender, marital status, residence, education level, ethnic group, and health insurance status.

c Model 3 adjusted smoking, alcohol drinking, social participation, chronic diseases, multimorbidity, self-rated health status, disability, BMI, ADL, and baseline intrinsic capacity.

**Figure 3:**
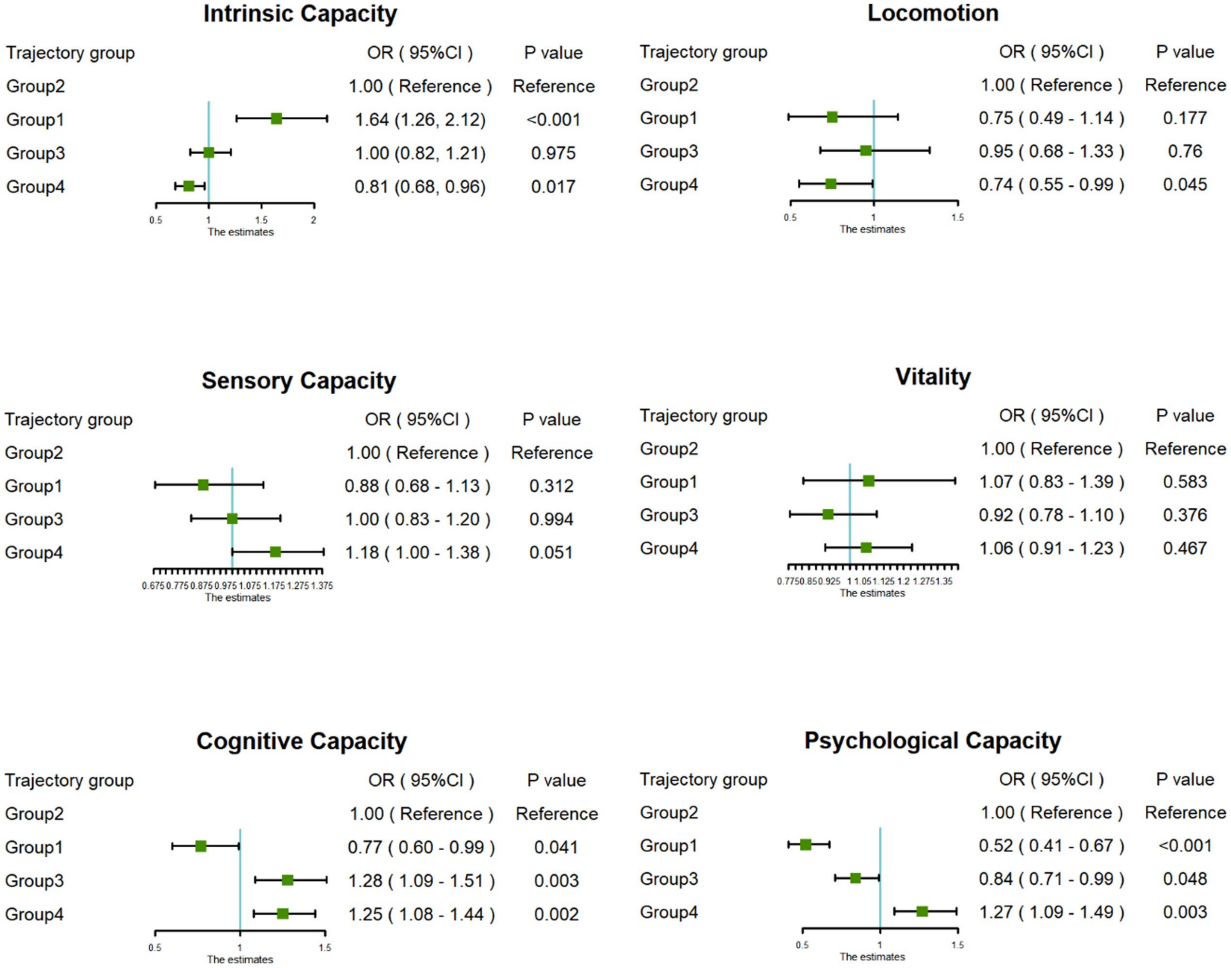
Forest plot of the associations between trajectory groups and dimensions of intrinsic capacity.

This study further explored the association between joint development trajectories and five subdomains of intrinsic capacity, and visualized the results of the association analysis in Figure 3. Compared with the “Continuous moderate nighttime sleep without napping” trajectory group, the “Persistent short nighttime sleep without napping” trajectory group had significantly lower cognitive capacity (OR = 0.77, 95% CI: 0.60–0.99) and psychological capacity (OR = 0.52, 95% CI: 0.41–0.67). The “Double moderate sleep duration” trajectory group showed higher cognitive capacity (OR = 1.25, 95% CI: 1.08–1.44) and psychological capacity (OR = 1.27, 95% CI: 1.09–1.49). In addition, the “Persistent short nighttime sleep with moderate napping” trajectory group showed higher cognitive capacity (OR = 1.28, 95% CI: 1.09–1.51).

### 3.4 Subgroup analysis

To assess the robustness of the association between joint developmental trajectories and intrinsic capacity, this study conducted subgroup analyses based on factors such as age, gender, residence, smoking, BMI, and multimorbidity. The results revealed that there were no significant differences in the strength and direction of the association between joint developmental trajectories and intrinsic capacity in each subgroup (P for interaction > 0.05), indicating that this association has good robustness and consistency in different populations with different characteristics. The detailed results of the subgroup analysis are shown in Table 4.

**Table 4:** Subgroup analyses by age, gender, residence, smoking, BMI, and multimorbidity.

Subgroup	Intrinsic capacity, OR (95%CI)				*P* for interaction
	Group 2	Group 1	Group 3	Group 4	
Age					0.858
< 60 (*n* = 3,321)	1.00 (Reference)	2.58 (1.87, 3.57)	1.17 (0.92, 1.49)	0.67 (0.53, 0.83)	
≥ 60 (*n* = 2,297)	1.00 (Reference)	2.45 (1.76, 3.41)	1.27 (0.99, 1.62)	0.74 (0.59, 0.93)	
Gender					0.376
Female (*n* = 2,657)	1.00 (Reference)	3.06 (2.30, 4.07)	1.50 (1.19, 1.89)	0.81 (0.65, 1.01)	
Male (*n* = 2,961)	1.00 (Reference)	2.17 (1.48, 3.17)	1.17 (0.92, 1.50)	0.72 (0.58, 0.90)	
Residence					0.378
Urban (*n* = 2026)	1.00 (Reference)	2.00 (1.31, 3.05)	1.26 (0.93, 1.70)	0.69 (0.52, 0.92)	
Rural (*n* = 3,592)	1.00 (Reference)	3.13 (2.38, 4.11)	1.38 (1.13, 1.70)	0.78 (0.65, 0.94)	
Smoking					0.117
No (*n* = 3,727)	1.00 (Reference)	3.07 (2.36, 4.01)	1.51 (1.23, 1.85)	0.84 (0.69, 1.02)	
Yes (*n* = 1891)	1.00 (Reference)	2.09 (1.34, 3.24)	1.02 (0.75, 1.37)	0.61 (0.47, 0.79)	
BMI					0.865
Underweight (*n* = 308)	1.00 (Reference)	3.35 (1.52, 7.37)	0.91 (0.48, 1.72)	0.81 (0.45, 1.46)	
Normal weight (*n* = 2,929)	1.00 (Reference)	2.69 (1.99, 3.63)	1.41 (1.13, 1.76)	0.78 (0.63, 0.96)	
Overweight (*n* = 1703)	1.00 (Reference)	2.63 (1.66, 4.17)	1.30 (0.93, 1.81)	0.72 (0.53, 0.98)	
Obese (*n* = 678)	1.00 (Reference)	2.42 (1.12, 5.21)	1.62 (0.93, 2.82)	0.96 (0.58, 1.59)	
Multimorbidity					0.876
None (*n* = 1847)	1.00 (Reference)	2.21 (1.34, 3.64)	0.99 (0.69, 1.40)	0.70 (0.52, 0.93)	
1–2 (*n* = 2,797)	1.00 (Reference)	2.26 (1.66, 3.08)	1.19 (0.94, 1.51)	0.74 (0.60, 0.92)	
3–5 (*n* = 916)	1.00 (Reference)	3.38 (2.04, 5.60)	1.38 (0.94, 2.01)	0.82 (0.56, 1.21)	
≥ 6 (*n* = 58)	1.00 (Reference)	1.20 (0.16, 8.80)	1.46 (0.27, 7.82)	0.53 (0.10, 2.98)	

OR, Odds Ratio; CI, Confidence Interval.

### 3.5 Sensitivity analysis

The sensitivity analysis of this study further verified the robustness of the results. Intrinsic capacity was included as a continuous variable in the linear regression model (Supplementary Table S2), and the results of the analysis were highly consistent with the conclusions of the binary logistic regression in the main analysis (Table 3). At the same time, the results of the analysis based on inverse probability weighting (IPW) (Supplementary Table S3) also confirmed the results of the main analysis: compared with the reference group, the “Persistent short sleep at night without napping” trajectory group had a significantly higher risk of low intrinsic capacity (OR = 1.55, 95% CI: 1.19–2.02); the “Double moderate sleep duration” trajectory group was more likely to have high intrinsic capacity (OR = 0.83, 95% CI: 0.71–0.98).

## 4 Discussion

### 4.1 Identification and characteristics of four joint developmental trajectories of nighttime sleep duration and nap duration

This study successfully identified four statistically significant types of sleep joint developmental trajectories. Among them, the “Persistent short sleep at night without napping” trajectory group faced long-term nighttime sleep deprivation and lacked a napping compensation mechanism. This phenomenon may be related to factors such as sleep structure disorders caused by aging, chronic diseases, or changes in lifestyle habits. Although the “Continuous moderate nighttime sleep without napping” trajectory group did not have a napping habit, they maintained regular and moderate nighttime sleep. This phenomenon may be attributed to their healthy lifestyles and good physical functioning. The “Persistent short sleep at night with moderate naps” trajectory group compensates for insufficient nighttime sleep with naps, which alleviates the problem of insufficient total sleep time to a certain extent. However, long-term insufficient nighttime sleep may still associate with cumulative health risks. Therefore, this group needs to focus on improving the quality of nighttime sleep and avoid over-reliance on naps to compensate. The “Double moderate sleep duration” trajectory group presented an ideal pattern of nighttime sleep and nap duration within the appropriate range. This sleep pattern may be beneficial for maintaining the physical function and delaying the aging process.

### 4.2 A sleep pattern characterized by consistently short nighttime sleep without naps is significantly associated with a risk of low intrinsic capacity

Good sleep quality has been recognized as an important protective factor for intrinsic capacity (21, 41). Previous studies (23, 25, 30) have shown that nighttime sleep duration is related to intrinsic capacity in a U-shaped curve. Chen et al. (23) considered that napping habits may have a different effect on intrinsic capacity than nighttime sleep duration does and reported that napping for ≤60 min has a protective effect against the risk of intrinsic capacity decline. However, these studies did not consider the synergistic development of nighttime sleep duration and nap duration. On this basis, this study takes a dynamic trajectory perspective to systematically investigate the impact of the joint developmental trajectory of nighttime sleep duration and nap duration on intrinsic capacity. We found that the “Persistent short sleep at night without napping” trajectory group has a significantly greater risk of low intrinsic capacity (OR = 1.64, 95% CI: 1.26–2.12). This sleep pattern mainly reduces the intrinsic capacity by affecting the cognitive capacity and psychological capacity dimensions of intrinsic capacity. The results of subgroup analysis and sensitivity analysis are consistent with this result. In-depth exploration of the underlying mechanisms suggests that sleep deprivation may adversely affect intrinsic capacity through multiple pathways, including impairing cognitive function by disrupting neurotransmitter rhythms (42), triggering a cascade of inflammatory responses that increase pro-inflammatory factors (43), and disrupting hormonal balance to affect metabolism and immune function (44).

Some research results differ from those of this study. Zhang et al. (21) reported that insufficient sleep was not significantly associated with a decline in intrinsic capacity. Chen et al. (23) reported that both excessive sleep duration (>10 h) and insufficient sleep duration (<6 h) have a negative effect on intrinsic capacity. These differences may stem from the fact that they measured nighttime sleep duration at a single point in time, whereas this study was based on multiple measurements. The study results emphasize the importance of considering nighttime sleep duration trajectories when conducting comprehensive assessments of older adults and provide more compelling evidence for research in this field.

### 4.3 Napping has a potential buffering effect on the risk of low intrinsic capacity caused by persistent short nighttime sleep

This study not only revealed the adverse effects of persistent short nighttime sleep on intrinsic capacity, but also further explored the potential compensatory effects of different nap patterns on nighttime sleep deprivation. Compared with the “Continuous moderate nighttime sleep without napping” trajectory group, the risk of low intrinsic capacity in the “Persistent short sleep at night with moderate napping” trajectory group did not show a statistically significant increase (OR = 1.00, 95% CI: 0.82–1.21). This result contrasts sharply with the significantly increased risk of low intrinsic capacity observed in the “Persistent short sleep at night without napping” trajectory group (OR = 1.64). This non-significant association (OR = 1.00) suggests that regular moderate napping may have a buffering or compensatory effect on the adverse effects associated with persistent nighttime sleep deprivation.

Although persistent short nighttime sleep is generally considered harmful to health, the results of this study suggest that regular moderate napping may partially offset the negative effects of short nighttime sleep alone through potential mechanisms such as restoring daytime alertness (45), alleviating sleep pressure (46), or promoting cognitive function recovery (47), thereby helping to maintain overall intrinsic capacity. In addition, this study further found that compared with the “Continuous moderate nighttime sleep without napping” trajectory group, middle-aged and elderly people in the “Persistent short nighttime sleep with moderate napping” trajectory group may exhibit higher cognitive function. The potential mechanism behind this phenomenon may be that although the people in this trajectory group had shorter nighttime sleep duration, their sleep efficiency was significantly higher, and efficient nighttime sleep can promote cognitive repair more fully. At the same time, moderate napping can further help restore and improve cognitive function by supplementing sleep duration.

The results of this study suggest that when assessing sleep health, it is important to consider the importance of the entire sleep pattern (including napping) rather than focusing solely on nighttime sleep. However, the results of this study should be interpreted with caution. The results only indicate that the risk was not significantly increased in the “Persistent short nighttime sleep with moderate napping” trajectory group, and it cannot be inferred that moderate napping has a clear protective effect against the harms of short nighttime sleep. The results are more likely to suggest a potential neutralizing or buffering effect, which prevented the risk level in this trajectory group from reaching the significant increase observed in the “Persistent short nighttime sleep without napping” trajectory group. Therefore, the exact mechanism of this compensatory effect and its universality in different subgroups remain to be verified by future studies using more accurate sleep measurement methods and a more in-depth analysis of the dimensions of intrinsic capacity.

### 4.4 A sleep pattern with moderate nighttime sleep and nap duration has a synergistic protective effect on intrinsic capacity

This study further analyzed the synergistic effects of different sleep duration combinations and found that compared with the “Continuous moderate nighttime sleep without napping” trajectory group, the “Double moderate sleep duration” trajectory group was more likely to exhibit high intrinsic capacity (OR = 0.81, 95% CI: 0.68–0.96). From a clinical perspective, this sleep pattern shows a reduction in the risk of low intrinsic capacity by approximately 19%, suggesting its potential value in maintaining high intrinsic capacity at the population level. This result suggests that, on the basis of maintaining moderate nighttime sleep (6–8 h), good napping habits (≤60 min) may maintain high intrinsic capacity through the following synergistic mechanisms: At the level of neuroplasticity, moderate sleep at night completes synaptic pruning and memory consolidation through a complete NREM-REM cycle, whereas naps promote offline memory replay by supplementing NREM slow-wave activity, reducing the hippocampus’s dependence on the neocortex for memory storage and improving task accuracy by 20% (48). In terms of metabolism and immune regulation, nighttime sleep maintains the circadian rhythm of leptin/ghrelin, whereas naps reduce daytime cortisol peaks by 28% by suppressing sympathetic nervous system activity (49) and decreasing the release of the pro-inflammatory factors IL-6 and TNF-*α* (50), thereby alleviating the damage caused by systemic inflammation to cognitive function. In addition, at the circadian rhythm synchronization level, nighttime sleep ensures the periodic expression of core clock genes, whereas naps serve as secondary circadian rhythm anchors, enhancing the circadian amplitude of serotonin and dopamine, thereby maintaining emotional stability and motivation levels (51). This finding provides a new perspective on healthy aging and sleep. Based on ensuring adequate nighttime sleep (6–8 h), scientifically planning moderate naps (≤60 min) may be a feasible intervention target for improving functional reserve in middle-aged and older adults.

## 5 Strengths and limitations

This study is the first to dynamically track the joint developmental trajectory of nighttime sleep duration and nap duration in middle-aged and older adults and systematically reveals the mechanism by which the interaction between the two sleep patterns in the time dimension affects intrinsic capacity, providing an innovative theoretical basis for the construction of a “dual-mode sleep” intervention strategy. The results of this study overcome the limitations of traditional single-mode sleep research and expand the research dimension of sleep health by integrating the dynamic association between nighttime sleep and nap duration. However, this study also has several limitations. First, the assessment of sleep duration in this study relies on participants’ self-reported data, which inevitably introduces risks of recall bias and subjective judgment errors. Due to limitations in equipment and resources, this study lacks the use of gold standard objective methods (such as actigraphy, polysomnography) to calibrate or validate these subjective data. Additionally, this study did not include metric of sleep quality or the presence of sleep disorders (such as insomnia, obstructive sleep apnea), which limits the interpretability of the results. Future studies should incorporate objective monitoring devices to enhance the reliability of the results. Second, this study was based on observational data, which, although it controlled for some confounding factors, could not completely rule out the interference of unmeasured variables (such as genetic factors and occupational history). Additionally, the study population was Chinese, and sleep habits may be influenced by regional cultural factors. Therefore, caution is advised when extrapolating conclusions to other cultural contexts. Further cross-cultural comparative studies are recommended to validate the universality of the “dual-mode sleep” theory proposed in this study.

## 6 Conclusion

This study systematically identified four joint development trajectories of nighttime sleep duration and nap duration in middle-aged and older adults. It found that middle-aged and older adults with consistently short nighttime sleep and no naps had a significantly increased risk of low intrinsic capacity, whereas moderate napping had a potential buffering effect on the risk of low intrinsic capacity caused by consistently short nighttime sleep. In addition, a “double moderate sleep pattern” with synergistic optimization of nighttime sleep duration and nap duration is an ideal strategy for maintaining the intrinsic capacity. The results of this study reveal the association between sleep duration development trajectories and intrinsic capacity, providing new theoretical references for optimizing “dual-mode sleep” management and developing targeted health intervention strategies for middle-aged and older adults.

## Data Availability

Publicly available datasets were analyzed in this study. This data can be found at: https://charls.pku.edu.cn.
