# Supplementary material for: The association between sleep duration trajectories and intrinsic capacity in middle-aged and older adults in China: a longitudinal Chinese study assessing healthy aging
Source: Front Med (Lausanne). 2025 Jul 7;12:1595241. doi: 10.3389/fmed.2025.1595241 (PMC12277252; doi:10.3389/fmed.2025.1595241)
Supplement: Supplementary file 1 [file Table_1.DOCX]

**Table S1. Baseline characteristics of included participants and excluded participants due to missing data on nighttime sleep duration, nap duration, and intrinsic capacity.**

| **Variables** | **Total (n = 13154)** | **Excluded (n = 7536)** | **Included (n = 5618)** | **Statistic** | ***P*** |
| --- | --- | --- | --- | --- | --- |
|  |  |  |  |  |  |
| Age, Mean ± SD | 59.02 ± 9.26 | 59.90 ± 9.90 | 57.85 ± 8.18 | t=12.98 | <0.001 |
| Gender, n(%) |  |  |  | χ²=88.96 | <0.001 |
| Female | 6847 (52.05) | 4190 (55.60) | 2657 (47.29) |  |  |
| Male | 6307 (47.95) | 3346 (44.40) | 2961 (52.71) |  |  |
| Marital Status, n(%) |  |  |  | χ²=68.40 | <0.001 |
| Others | 1544 (11.75) | 1035 (13.76) | 509 (9.06) |  |  |
| Married | 11598 (88.25) | 6489 (86.24) | 5109 (90.94) |  |  |
| Residence, n(%) |  |  |  | χ²=0.11 | 0.746 |
| Urban | 4723 (35.91) | 2697 (35.79) | 2026 (36.06) |  |  |
| Rural | 8431 (64.09) | 4839 (64.21) | 3592 (63.94) |  |  |
| Education level, n(%) |  |  |  | χ²=168.13 | <0.001 |
| Primary school or below | 8956 (68.23) | 5465 (72.79) | 3491 (62.14) |  |  |
| Junior high school or above | 4170 (31.77) | 2043 (27.21) | 2127 (37.86) |  |  |
| Ethnic group, n(%) |  |  |  | χ²=50.22 | <0.001 |
| Others | 890 (7.38) | 577 (8.95) | 313 (5.57) |  |  |
| Han Chinese | 11173 (92.62) | 5868 (91.05) | 5305 (94.43) |  |  |
| Health insurance, n(%) |  |  |  | χ²=10.96 | <0.001 |
| No | 769 (5.89) | 482 (6.49) | 287 (5.11) |  |  |
| Yes | 12279 (94.11) | 6948 (93.51) | 5331 (94.89) |  |  |
| Smoking, n(%) |  |  |  | χ²=66.23 | <0.001 |
| No | 8914 (70.06) | 5187 (72.99) | 3727 (66.34) |  |  |
| Yes | 3810 (29.94) | 1919 (27.01) | 1891 (33.66) |  |  |
| Alcohol drinking, n(%) |  |  |  | χ²=45.90 | <0.001 |
| No | 8709 (66.54) | 5152 (68.96) | 3557 (63.31) |  |  |
| Yes | 4380 (33.46) | 2319 (31.04) | 2061 (36.69) |  |  |
| Social participation, Mean ± SD | 1.30 ± 1.79 | 1.13 ± 1.68 | 1.53 ± 1.91 | t=-12.24 | <0.001 |
| Hypertension, n(%) |  |  |  | χ²=10.52 | 0.001 |
| No | 9703 (74.33) | 5447 (73.25) | 4256 (75.76) |  |  |
| Yes | 3351 (25.67) | 1989 (26.75) | 1362 (24.24) |  |  |
| Diabetes, n(%) |  |  |  | χ²=1.92 | 0.166 |
| No | 12247 (94.03) | 6946 (93.78) | 5301 (94.36) |  |  |
| Yes | 778 (5.97) | 461 (6.22) | 317 (5.64) |  |  |
| Cancer, n(%) |  |  |  | χ²=0.85 | 0.357 |
| No | 12939 (99.15) | 7364 (99.09) | 5575 (99.23) |  |  |
| Yes | 111 (0.85) | 68 (0.91) | 43 (0.77) |  |  |
| Heart disease, n(%) |  |  |  | χ²=2.48 | 0.115 |
| No | 11484 (88.04) | 6509 (87.65) | 4975 (88.55) |  |  |
| Yes | 1560 (11.96) | 917 (12.35) | 643 (11.45) |  |  |
| Stroke, n(%) |  |  |  | χ²=22.62 | <0.001 |
| No | 12752 (97.53) | 7231 (96.97) | 5521 (98.27) |  |  |
| Yes | 323 (2.47) | 226 (3.03) | 97 (1.73) |  |  |
| Mental disorder, n(%) |  |  |  | χ²=7.48 | 0.006 |
| No | 12864 (98.47) | 7313 (98.21) | 5551 (98.81) |  |  |
| Yes | 200 (1.53) | 133 (1.79) | 67 (1.19) |  |  |
| Cognitive impairment, n(%) |  |  |  | χ²=22.57 | <0.001 |
| No | 12846 (98.32) | 7288 (97.85) | 5558 (98.93) |  |  |
| Yes | 220 (1.68) | 160 (2.15) | 60 (1.07) |  |  |
| Multimorbidity, n(%) |  |  |  | χ²=14.55 | 0.002 |
| None | 4148 (31.66) | 2301 (30.75) | 1847 (32.88) |  |  |
| 1-2 | 6528 (49.83) | 3731 (49.87) | 2797 (49.79) |  |  |
| 3-5 | 2254 (17.21) | 1338 (17.88) | 916 (16.30) |  |  |
| ≥6 | 170 (1.30) | 112 (1.50) | 58 (1.03) |  |  |
| Self-rated health status, n(%) |  |  |  | χ²=135.80 | <0.001 |
| Unhealthy | 3604 (27.53) | 2352 (31.48) | 1252 (22.29) |  |  |
| Healthy | 9486 (72.47) | 5120 (68.52) | 4366 (77.71) |  |  |
| Disability, n(%) |  |  |  | χ²=37.03 | <0.001 |
| No | 12552 (95.82) | 7100 (94.89) | 5452 (97.05) |  |  |
| Yes | 548 (4.18) | 382 (5.11) | 166 (2.95) |  |  |
| BMI, n(%) |  |  |  | χ²=46.41 | <0.001 |
| underweight | 738 (6.85) | 430 (8.33) | 308 (5.48) |  |  |
| normal weight | 5702 (52.90) | 2773 (53.73) | 2929 (52.14) |  |  |
| overweight | 3099 (28.75) | 1396 (27.05) | 1703 (30.31) |  |  |
| obese | 1240 (11.50) | 562 (10.89) | 678 (12.07) |  |  |
| ADL, Mean ± SD | 0.33 ± 0.94 | 0.43 ± 1.11 | 0.19 ± 0.62 | t=15.99 | <0.001 |
| Intrinsic capacity, n(%) |  |  |  | χ²=1145.53 | <0.001 |
| High | 7453 (58.58) | 3228 (45.44) | 4225 (75.20) |  |  |
| Low | 5269 (41.42) | 3876 (54.56) | 1393 (24.80) |  |  |
| t: t-test, χ²: Chi-square test | | | | | |
| SD: standard deviation | | | | | |

**Table S2. Association between trajectory groups and intrinsic capacity (continuous variable).**

| **Trajectory Group** | **Model 1^a^**  **β (95% CI)** | **Model 2^b^**  **β (95% CI)** | **Model 3^c^**  **β (95% CI)** |
| --- | --- | --- | --- |
| Group 2 | 0.00 (Reference) | 0.00 (Reference) | 0.00 (Reference) |
| Group 1 | -0.64 (-0.77, -0.51) | -0.45 (-0.57, -0.34) | -0.21 (-0.31, -0.10) |
| Group 3 | -0.19 (-0.28, -0.10) | -0.13 (-0.21, -0.05) | 0.01 (-0.06, 0.08) |
| Group 4 | 0.15 (0.08, 0.23) | 0.14 (0.07, 0.21) | 0.08 (0.02, 0.14) |
| β = beta; CI = Confidence Interval | | | |
| a: Model 1 did not adjust any variables | | | |
| b: Model 2 adjusted for age, gender, marital status, residence, education level, ethnic group, and health insurance status | | | |
| c: Model 3 adjusted smoking, alcohol drinking, social participation, chronic diseases, multimorbidity, self-rated health status, disability, BMI, ADL, and baseline intrinsic capacity | | | |

**Table S3. Association between trajectory groups and intrinsic capacity using inverse probability weighting.**

| **Trajectory Group** | **Main Analysis^a^: Multivariable-Adjusted Model**  **OR (95% CI)** | **Sensitivity Analysis^b^: IPTW Model**  **OR (95% CI)** |
| --- | --- | --- |
| Group 2 | 1.00 (Reference) | 1.00 (Reference) |
| Group 1 | 1.64 (1.26, 2.12) | 1.55 (1.19, 2.02) |
| Group 3 | 1.00 (0.82, 1.21) | 1.07 (0.90, 1.27) |
| Group 4 | 0.81 (0.68, 0.96) | 0.83 (0.71, 0.98) |
| IPTW = Inverse Probability Treatment Weighting; OR = Odds Ratio; CI = Confidence Interval | | |
| a. Main analysis adjusted for smoking, alcohol drinking, social participation, chronic diseases, multimorbidity, self-rated health status, disability, BMI, ADL, and baseline intrinsic capacity | | |
| b. Sensitivity analysis used stabilized inverse probability treatment weights truncated at 1st and 99th percentiles | | |
